# Supplementary figures and images for: Vision guides the twilight search for oviposition sites of the Asian tiger mosquito, Aedes albopictus
Source: PLoS Negl Trop Dis. 2024 Nov 27;18(11):e0012674. doi: 10.1371/journal.pntd.0012674 (PMC11602101; doi:10.1371/journal.pntd.0012674)

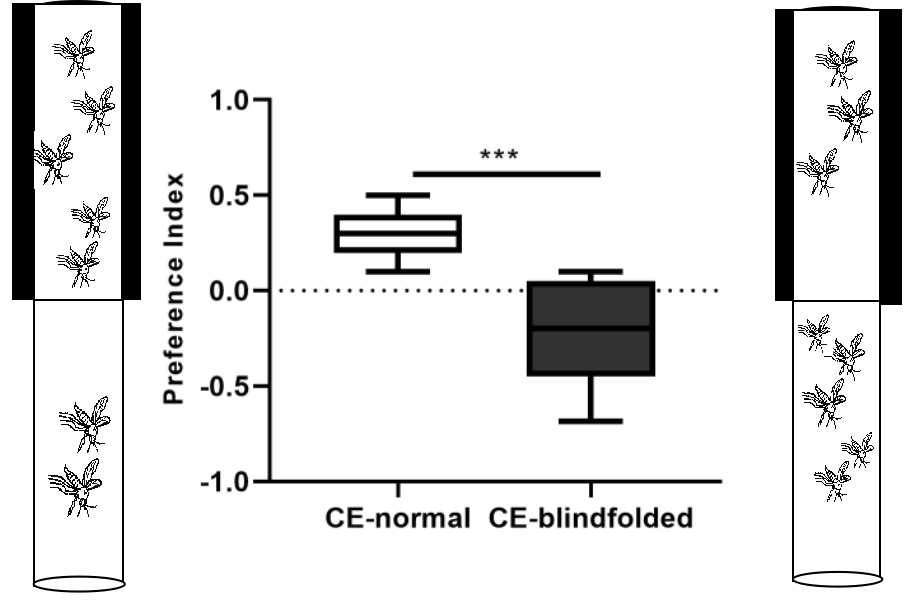

Supplement: S1 Fig — Comparison of the shadow index of applied compound eyes with wild-type gravid mosquitoes. CE-blindfolded is the use of acrylic paint to apply mosquito compound eyes. shadow index = (number of mosquitoes in black environment—number of mosquitoes in light environment)/total. (t-test, t = 5.039, ***P<0.001, n = 9, Error bar represent mean ± SEM). (TIF) [file pntd.0012674.s001.tif]

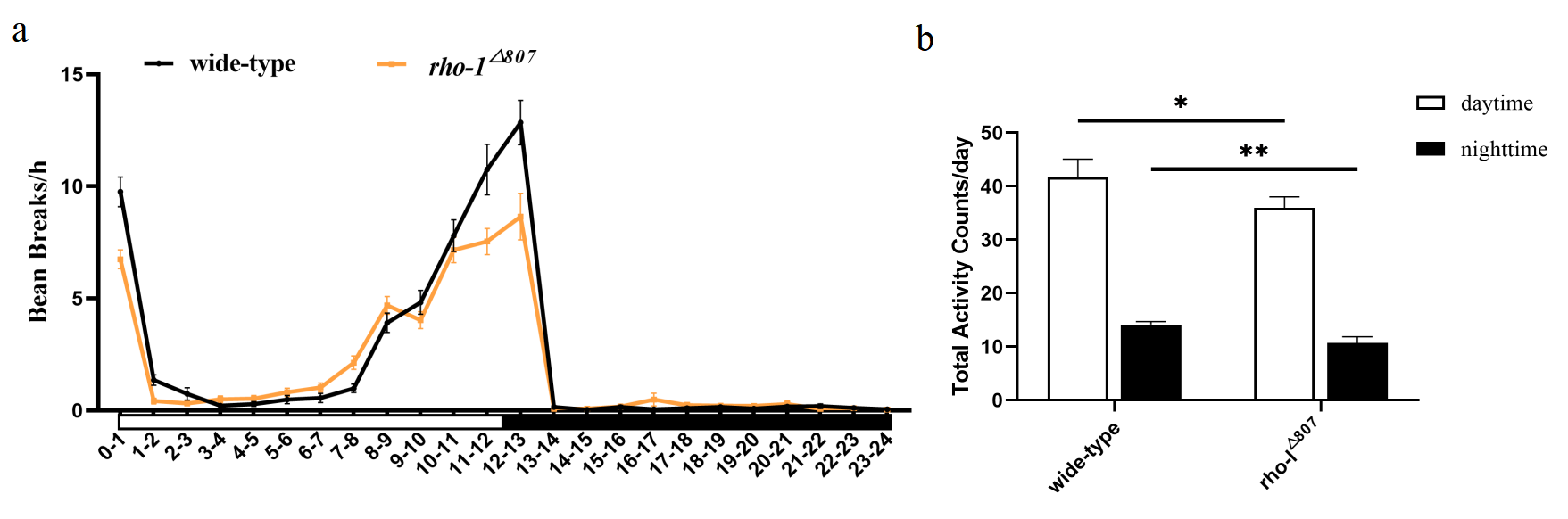

Supplement: S2 Fig — (a) Line graphs of mean activity of Aedes albopictus wild-type strain and rho-1Δ807 strain under LD light conditions for 4 consecutive days (days 4 to 7), white bars represent daytime and black bars represent nighttime; (b) Comparison of daytime and nighttime activity of Aedes albopictus wild-type strain and rho-1Δ807 strain under LD light conditions, Aedes albopictus wild-type, n = 29. rho-1Δ 807 strain, n = 28. (t-test, *P<0.05, **P<0.01, Error bar represent mean ± SEM). (TIF) [file pntd.0012674.s002.tif]

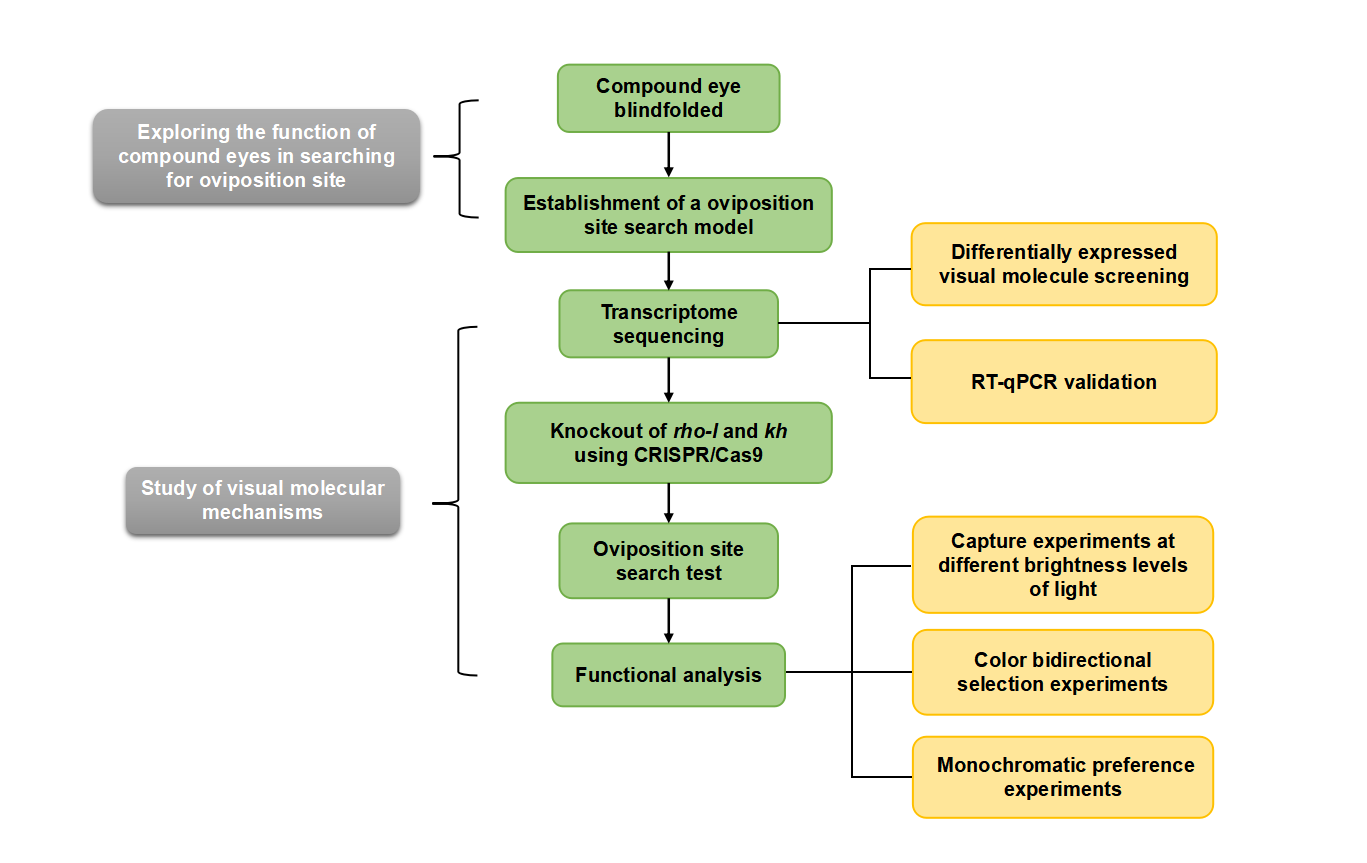

Supplement: S3 Fig — (TIF) [file pntd.0012674.s003.tif]
