# Supplementary material for: Vision guides the twilight search for oviposition sites of the Asian tiger mosquito, Aedes albopictus
Source: PLoS Negl Trop Dis. 2024 Nov 27;18(11):e0012674. doi: 10.1371/journal.pntd.0012674 (PMC11602101; doi:10.1371/journal.pntd.0012674)
Supplement: S1 Table — (DOCX) [file pntd.0012674.s004.docx]

S1 Table. Amino acid sequences and base sequences of wild-type mosquitoes and mutant strains.

| Gene name | Sequences |
| --- | --- |
| *rho-l* | MLGEPLAPMPLSWISTAAVSNSNVTVVDRVPPEMLYMVDAHWYQFPPLNPLWHSILGFAIFVLACVSVCGNGMVMYIFTTTKPLRTPSNMMIINLAFSDFLMMFTMGPPMIINCYNETWVFGPFACEVYGMLGSLSGCVSIWSMTMIAFDRYNVIVKGLSAKPMTFNGSLLKILFVWINSLFWTLAPMFGWNRYVPEGNMTACGTDYLSQDIVSRSYIMVYSFFVYWFPLLMIIYSYTFILKAVSEHEKNMREQAKKMNVSSLRSSDAAKQSAEIKLAKVALVTISLWFMAWTPYLVINYAGIFNTAPISPLATIWGSLFAKANAVYNPIVYGISHPKYRAALYQRFPSLACATETPAA |
| *rho-l^△807^* | MLGEPLAPMPLSWISTAAVSNSNVTVVDRVPPEMLYMVDAHWYQFPPLNPLWHSILGFAIFVLACVSVCGNGMVMYIFTTTKPLRTPSNMMIINLAFSDFLMMFTMGPPMIINCYNETSLPRHQLCRNLQHSAD*PTHHLGVAVRQGQRRVQSHSLRYQPSQVSCCPVPAIPITGLRNGDTSSSSR**PVCCISGNRCIGSCEQCL |
| *rho-l* | ATGTTGGGAGAACCATTAGCCCCGATGCCGTTATCGTGGATTAGTACAGCAGCAGTATCCAACAGCAATGTGACAGTGGTCGATAGGGTTCCACCGGAAATGTTGTACATGGTGGATGCGCACTGGTATCAGTTTCCGCCACTCAATCCATTGTGGCACTCAATACTCGGATTTGCCATCTTCGTACTCGCGTGTGTTTCAGTCTGTGGAAACGGAATGGTCATGTACATTTTCACAACAACCAAGCCGCTTCGCACTCCATCCAACATGATGATCATCAACTTGGCCTTTTCCGATTTCCTCATGATGTTCACTATGGGCCCACCAATGATAATCAACTGCTACAACGAAACCTGGGTCTTCGGGCCATTCGCATGTGAAGTGTACGGAATGCTGGGCTCATTGTCCGGTTGCGTCTCGATCTGGAGTATGACAATGATCGCCTTCGACCGATACAATGTGATAGTGAAGGGCCTTTCGGCTAAACCGATGACCTTTAATGGATCGTTGCTGAAGATTTTGTTCGTTTGGATCAATTCGCTGTTCTGGACTTTGGCTCCGATGTTCGGATGGAACCGATACGTACCAGAAGGGAATATGACTGCCTGCGGTACGGACTATCTTAGCCAAGATATTGTCAGCCGGTCCTACATTATGGTGTACTCGTTCTTTGTCTACTGGTTTCCGTTGCTAATGATCATCTACTCCTACACATTCATTTTGAAGgtaagatgtttcttcaatctttttcttcgatttactttttatgtatattattttacaaatcttttgcagtgaaaatttaatgtgtttcttgatttgtttcttgttcatcctaaattttgttatcatgcgtttgattatttcattcgcaatttcatgaaacatttcaatacgtagataatgatgacttacgcagcggtttacactgttcagtaaatccccacattattggttttggtaatagtgcaaaagtcttaaaaactatcctacctcaatcttcctgcagGCCGTTTCCGAGCATGAGAAGAACATGCGCGAACAAGCCAAAAAGATGAACGTGTCATCGCTCCGATCATCGGATGCGGCCAAACAAAGCGCGGAAATCAAACTGGCCAAGGTGGCCCTGGTTACCATCTCGCTGTGGTTCATGGCCTGGACTCCCTACCTCGTCATCAACTATGCCGGAATCTTCAACACAGCGCCGATTAGCCCACTGGCCACCATCTGGGGGTCGCTGTTCGCCAAGGCCAACGCCGTGTACAATCCCATAGTCTACGGTATCAGCCATCCCAAGTATCGTGCTGCCCTGTACCAGCGATTCCCATCACTGGCCTGCGCAACGGAGACACCAGCAGCAGCAGCCGGTGATGACCAGTCTGTTGCATCAGCGGGAACCGTTGCATCGGATCCTGCGAACAGTGCTTAA |
| *rho-l^△807^* | GCGCAGCTGCAGTCGACGATTTAAGGCACTGTTCGCAGGATCCGATGCAACGGTTCCCGCTGATGCAACAGACTGGTCATCAGCGGCTGCTGCTGCTGGTGTCTCCGTTGCGCAGGCCAGTGATGGGAATCGCTGGTACAGGGCAGCACGATACTTGGGATGGCTGATACCGTAGACTATGGGATTGTACACGGCGTTGGCCTTGGCGAACAGCGACCCCCAGATGGTGGCCAGTGGGCTAATCGGGGCTGTGTTGAAGATTCCGGCATAGTTGATGACGAGGTAGGGAGGTTTCGTTGTAGCAGTTGATTATCATAGGTGGGCCCATAGTGAACATCATGAGGAAATCGGAAAATGCCAAGTTGATGATCATCATGTTGGATGGAGTGCGAAGCGGTTTGGTTGTTATGAAAATATACATGACCATTCCGTTACCACAGATCGAAACACACGCGAGTACGAAGATGGCAAATCCGAGTATTGAGTGCCACAATGGATTGAGTGGCGGAAACTGATACCAGTGCGCATCCACCATGTACAACATTTCCGGTGGAACCCTATCGACCACTGTCACATTGCTGTTGGATACTGCTGCTGTACTAATCCACGATAATGGCATCGGAGCAAATGGTTCTCCCAACATTTTTTGTCACAAATGCACTGTCTCAGCTTTTCAGCAACGGTTTACAGATTTTTAAGAAACACACCACTTAGATCTCTAATCCTTGTGAGCTGATTTTTGGGTAATCCTTCGATGGGTTTATTCCGGGTCTACCTTGACGAATCTCGAGAGCGACTTGAACCCCAACCTTGCATCTCTAGAGGATCCCCGGGTACCGAGCTCGAATTCGTAATCATGGTCATAGCTGTTTCCTGTGTGAAATTGTTATCCGCTCACAATTCCACACAACATACGAGCCGGAAGCATAAAGTGTAAAGCCTGGGGTGCCTAATGAGTGAGCTAACTCACATTAATTGCGTTGCGCTCACTGCCCCGCTTTCCAGTCGGGAAACTTGTCGTGCCAGCTGTCAT |
