# Supplementary material for: Vision guides the twilight search for oviposition sites of the Asian tiger mosquito, Aedes albopictus
Source: PLoS Negl Trop Dis. 2024 Nov 27;18(11):e0012674. doi: 10.1371/journal.pntd.0012674 (PMC11602101; doi:10.1371/journal.pntd.0012674)
Supplement: S2 Table — (DOCX) [file pntd.0012674.s005.docx]

S2 Table. List and sequence of oligonucleotide primers used in this study.

| Primer name | Sequences (5’-3’) | Primer use |
| --- | --- | --- |
| sgRNA-F1 | GAAATTAATACGACTCACTATA TGCGAATGGCCCGAAGACCC GTTTTAGAGCTAGAAATAGC | sgRNA1 synthesis of *rho-l* |
| sgRNA-F2 | GAAATTAATACGACTCACTATA GATGACGAGGTAGGGAGTCC GTTTTAGAGCTAGAAATAGC | sgRNA2 synthesis of *rho-l* |
| sgRNA-R | AAAAGCACCGACTCGGTGCCACTTTTTCAAGTTGATAACGGACTAGCCTTATTTTAACTTGCTATTTCTAGCTCTAAAAC | CRISPR R synthesis of *rho-l* |
| Aal-F | GGGGTTCAAGTCGCTCTCAA | Genotype identification for *rho-l* with PCR |
| Aal-R | ACGAATTAAGGCACTGTTCGC | Genotype identification for *rho-l* with PCR |
| *rho-l*-Fw-qPCR | CGTGTCATCGCTCCGATCAT | Expression profile analysis for *rho-l* with qPCR |
| *rho-l*-Rv-qPCR | GGCGCTGTGTTGAAGATTCC | Expression profile analysis for *rho-l* with qPCR |
| AAL-β-actin-qPCR-Fw | GCCGTCTTCCCGTCCATC | Reference gene for *rho-l* control with qPCR |
| AAL-β-actin-qPCR-Rv | GGCGACACGCAGCTCATT | Reference gene for *rho-l* control with qPCR |
| AAL-kh-Fw-qPCR | ATCAAGTGCAAGCCGTACAAC | Expression profile analysis for *Aalkh* with qPCR |
| AAL-kh-Rv-qPCR | ACAATCCTCAAACCCGGCAT | Expression profile analysis for *Aalkh* with qPCR |
