# Supplementary material for: Vision guides the twilight search for oviposition sites of the Asian tiger mosquito, Aedes albopictus
Source: PLoS Negl Trop Dis. 2024 Nov 27;18(11):e0012674. doi: 10.1371/journal.pntd.0012674 (PMC11602101; doi:10.1371/journal.pntd.0012674)
Supplement: S3 Table — (DOCX) [file pntd.0012674.s006.docx]

S3 Table. Statistics of mutation rates of G_0_ adults in *Ae. albopictus*.

| Group | Embryos injected | Hatched (%) | Pupated (%) | G_0_ adult survivors (%) | G_0_ mosaic (%) |
| --- | --- | --- | --- | --- | --- |
| *Ae. albopictus rho-l* | 340 | 15.50%  （53/340） | 90.05%  （48/53） | 97.90%  （47/48） | 8.50%  （4/47） |
