# Supplementary material for: Vision guides the twilight search for oviposition sites of the Asian tiger mosquito, Aedes albopictus
Source: PLoS Negl Trop Dis. 2024 Nov 27;18(11):e0012674. doi: 10.1371/journal.pntd.0012674 (PMC11602101; doi:10.1371/journal.pntd.0012674)
Supplement: Attachment — Submitted filename: 20241004 edited_Responses to reviewers 2.1.docx [file pntd.0012674.s009.docx]

**Dear Editors and Reviewers:**

Thank you for your letter and for the reviewers’ comments concerning our manuscript entitled “Vision guides the twilight search for oviposition sites of the Asian tiger mosquito, *Aedes albopictus*”. Those comments are all valuable and very helpful for revising and improving our paper, as well as the important guiding significance to our researches. We have studied comments carefully and have made correction which we hope meet with approval.

Reviewer #1:
1. Transcriptome sequencing, the purpose of this is not clear, to identify differentially expressed visual–related genes or to examine odor-related genes? The purpose of this experiment will determine the control and test groups. The purpose of this experiment needs to be clarified.

**Response:** Transcriptome sequencing was used to analyze visual genes associated with oviposition site search behavior in *Aedes albopictus*. The test and control groups were carried out as below:

Test group: One hundred of female mosquitoes were blindfolded on their compound eyes 48 hours after blood feeding, then released into a 1m^3^ mosquito net. Two three-hole oviposition traps were placed diagonally in the net, one containing 100 ml of deionized water and the other empty. Heads of female mosquitoes that flew into the three-hole Mosq-ovitrap with water were collected and stored in TRIZOL, then 30 individuals were pooled for sequencing.

Control group: One hundred of female mosquitoes without compound eye blindfolded treatment were kept in the same rearing environment and batch as the test group. They were released into a 1m^3^ mosquito net 72 hours after blood feeding. Two three-hole oviposition traps were placed diagonally in the net, one containing 100 ml of deionized water and the other empty. Heads of female mosquitoes that flew into the three-hole Mosq-ovitrap with water were collected and stored in TRIZOL, then 30 individuals were pooled for sequencing. Transcriptome sequencing data from both groups will be analyzed to identify differentially expressed genes associated with oviposition site search behavior, with particular focus on visual genes. The time required to collect a replicate of 30 female mosquitoes differed between the experimental and control groups. Line 187-203.

2. Transcriptome sequencing: “Mosquitoes with blindfolded compound eyes flying into the three-hole Mosq-ovitrap will be the test group, while those with unblindfolded eyes flying into the three-hole Mosq-ovitrap device will be the control group”, is this correct? Or should it be “Mosquitoes with blindfolded compound

eyes (no visual cue) did not fly into the traps will be the test group, while those with unblindfolded eyes (has visual cue) flying into the traps will be the control group” if testing for visual-related genes?

**Response**: “Mosquitoes with blindfolded compound eyes flying into the three-hole Mosq-ovitrap will be the test group, while those with unblindfolded eyes flying into the three-hole Mosq-ovitrap device will be the control group” is correct, because this experiment will only evaluate the visual-related difference in searching oviposition site between the test group and the control group.

3. Similarly, the authors should describe the purpose of “CRISPR/Cas9 editing experiments of rho-l and kh genes” and for “Color bidirectional selection experiments”, so that readers have a better understanding of the context and to determine if the methods used are appropriate. In fact, the best way to do is to draw a flowchart to describe the flows of experimental design and the purpose of each experiment, although most studies lack this.

**Response**: We appreciate the reviewer’s feedback and have added a description of the purpose of the “CRISPR/Cas9 editing experiments for the rho-l and kh genes” (Line291-294) and the “color bi-directional choice experiment” (Line 318-323). The gene editing experiments were designed to investigate the role of *rho-l* and *kh* genes in oviposition site selection, while the color bi-directional choice experiment aimed to explore the factors influencing the selection of oviposition containers by mosquitoes and identify the genes associated with this behavior.

To further clarify the experimental objectives, a flowchart outlining the experimental design is provided in S3 Fig. Line 337-339.

4. Line 254: “The oviposition activity index is the number of eggs of other-colored oviposition mosquitoes - number of black oviposition mosquitoes)/ (number of eggs of other colored oviposition mosquitoes + number of black oviposition mosquitoes)”, why not simply use number of eggs of other-colored oviposition mosquitoes vs. number of black oviposition mosquitoes?

**Response**: This approach was implemented to simultaneously reduce non-specific selection and improve the precision and accuracy of the findings. The oviposition index (OVI) is a standard, two-way competitive index that assesses oviposition preference, with positive or negative values indicating attraction or avoidance, respectively. For clarity, we present a simplified formula: OVI = (Number of eggs in non-black control cups – Number of eggs in black cups) / Total number of eggs laid in the experiment. Line 331-333.

5. Line 301: “Statistical analysis”, “T-test” should be “t-test”, because usually “T-test” is used for “Holling T-test”

**Response**: We've changed the capital T to the lowercase t. Line 385.

6. Lines 311-317: This section should move to “Method” section, this describes the purpose of the method used especially the authors include the references here. In the “Result” section, only describe the results. Lines 331-332 may also be moved to “Method” section.

**Response**: We have addressed them by revising and refining the manuscript accordingly. We have moved the section (Line 397-399) to the Methods section (Line 139~141). 416-417 rows have been deleted.

7. In line 327, please use (b) and (d) instead of “b and d”.

**Response**: We have added parentheses. Line 414.

8. Lines 333-334: A statistical test result should be added here to describe the significance.

**Response**: We have incorporated the p-values and degrees of freedom into the manuscript, as suggested by the reviewer. Line 421.

**Response**: We have streamlined the manuscript by removing redundant sections. We added a sentence to clarify: “We built a model to record the search index (Fig 1b).”. Line 424~428.

10. Line 348: need a degree of freedom for the t-test. This occurred in many places in the results, please check all t-test results in the text, please use “t=???, d.f. = ?, P-value”, all tests need a degree of freedom.

**Response**: We have checked the results of the t-test tables throughout the paper.

Line436;453;526;550~551;561~562;588;590;607;610;613;662;668~669;677~678;680~681;693~694.

11. Lines 357-359: Please move this sentence to “Method” section.

**Response**: We have removed the redundant section, streamlining the manuscript. We have moved the section (Line 445-447) to the Methods section (Line 269~272).

12. Line 362: Need test method, statistical value, degree of freedom.

**Response**: We have made appropriate additions. The p-value here refers to the transcriptomic analysis. The negative binomial distribution was used for the statistical analysis. Genes with an adjusted p-value < 0.05 were considered differentially expressed as identified by DESeq2. Line 450.

13. Lines 367-373: Please move this section to “Methods” section.

**Response**: We made changes to the redundancy. We have moved the section (Line 455-458) to the Methods section (Line 260-263).

14. Line 408: Fig 2, please use “(d), (e), (f), and (g)”.

**Response**: We have added parentheses. Line 498.

15. Lines 411-425: Should this part be moved to method? It described the method rather than results.

**Response**: We have incorporated the additional information into the methods section and refined the results section for conciseness. Line 501~502.

16. Lines 435-443: Please move this part to “Methods” section.

**Response**: We removed the duplicates. Line 530~532.

17. Lines 469-476: Please move this part to " Methods” section.

**Response**: We have removed the redundancy. Line 570~577.

18. Lines 494-500: Please move this part to " Methods” section.

**Response**: We simplify the repetitive part of the method. “We used red, green, and blue LEDs to study the role of light spectra in oviposition site choice and the phenotypic differences in the *rho-l^△807^* strain”. Line 596~598.

19. Lines 509-518: This section should be described in the “Discussion” section.

**Response**: We incorporate this part into the discussion section. Line 723~728.

20. Lines 512-526: This belongs to the “Method” section.

**Response**: We removed the duplicates and kept the ones that needed to be kept. Line 626~627.

21. Lines 572-580: This section should be described in the “Discussion” section.

**Response**: We have removed the redundancy and moved the section to the “Discussion” section (Line 752-759).

22. Lines 588-592: This is the conclusion of the study, should be described at the end of the paper.
**Response**: This section was deemed redundant and has been removed. Line 698-702.

Reviewer #2: The work is of high scientific value and rigor.
1. The authors say they used a brush to coat the mosquito's compound eyes with a black water-soluble pigment, but they don't say how much was used. This should be specified.

**Response**: Thanks for your suggestion. We used 100 microliters for one mosquito. We have added this dosage into the Method. (Line 149~150)

2. Line 103 needs more literature support.

**Response**: We have added more references. Line111.

3.Line 358, change “should result in” to “will result in”.

**Response**: Done. Line270.

4. Line 525, change “forms different color patterns” to “and forms different color patterns”.

**Response**: Done. Line 628.

5. Line 657 needs more literature support.
**Response**: We have added more references. Line 783.

Reviewer #3:
Introduction:
~ Lines 76-78: The sentence here seems incomplete if the public health importance of these diseases are not emphasized.
**Response**: We have modified this sentence. Lines 76 to 79.
~ Lines 98-102: To improve readability, this sentence can be broken into two sentences.
**Response**: This sentence has been modified as “Each ommatidia can see a small part of the field and all of the view is combined to form a complete visual image of the mosquito (22, 23)”. Lines 104 to 106.

Methods:
~ Generally, apart from the locomotor activity section, no information was given on the age of the mosquitoes when they were used for the experiments. The only thing close was the days after blood-feeding that the mosquitoes were selected. Age seems to play a very significant role on several aspects of mosquito biology, so it would be important if the age of the mosquitoes were mentioned.

**Response**: Thanks for your suggestion. The blindfolded test used mosquitoes 2 days after the blood-feeding, while the search test used mosquitoes 3 days after the blood-feeding. The age of the mosquitoes has been supplemented in the Method accordingly. Line 135~137.

~ For the RNA-seq experiments (lines 168-170), was there any reason these controls were not considered?: (a) blindfolded compound eyes not flying into the three-hole Mosq-ovitrap and (b) unblindfolded compound eyes not flying into the three-hole Mosq-ovitrap device.

**Response**: Because the mosquitoes in these controls did not flying into the three-hole Mosq-ovitrap with water, therefore their compound eyes are not related with the oviposition site selection, that is why they are not considered.

~ Lines 254-258 - The equation is not easily comprehensible.

**Response**: The oviposition index (OVI) is a standard metric for comparing egg-laying rates, with values ranging from -1 to 1, indicating attraction or avoidance, respectively. To simplify the concept, we revised the formula as follows: OVI = (Number of eggs in non-black control cups – Number of eggs in black cups) / Total number of eggs laid in the experiment. Line331~333.

~ Lines 289-290 - The activity monitoring system has also been used in mosquitoes recently in the last five years: Ajayi et al 2022 (<https://doi.org/10.1242/jeb.244032>).

**Response**: We have specifically cited this reference in our work. Line374.

~ To address the issue of olfaction obscuring the effect of visual cues, the authors can do well by running specific experiments with mosquitoes showing mutations in olfactory responses (orco mutants etc).

**Response**: The search for oviposition sites is a combined sense process including vision, olfaction and gustation. As compound eye blindfolded only affects visual cues and not olfactory cues, this experimental design allowed us to specifically investigate the role of vision in oviposition site selection. The impact of olfactory cues on oviposition site selection has been previously explored in our published work *(Zhao et al*., *Insect Science*, 2024). The reference is cited on line 95.

Results:
~ SWISS-MODEL was mentioned once and there is was prior description about it. It will be helpful if you can provide details about the model whether in the methods or results section.

**Response**: We performed the prediction and analysis directly on the website (https://swissmodel.expasy.org/) and based on this analysis we mapped the amino acid structure of *rho-l*. We have added it to the methods section. Line309~314.

Discussion:
~ Lines 638-640 seems to be too short to be a paragraph.
Response: We rearranged the paragraphs. Line762~764.
